# Effect of blockchain technology initiatives on firms’ market value

**DOI:** 10.1186/s40854-023-00456-8

**Published:** 2023-02-01

**Authors:** Haji Suleman Ali, Feiyan Jia, Zhiyuan Lou, Jingui Xie

**Affiliations:** 1 School of Management, University of Science and Technology of China, Hefei, China; 2 School of Commerce and Accountancy, University of Management and Technology, Lahore, Pakistan; 3 College of Business, City University of Hong Kong, Kowloon Tong, Hong Kong; 4 Center for Digital Transformation, School of Management, Technical University of Munich, Heilbronn, Germany

**Keywords:** Blockchain, Operation efficiency, Time and cost saving, Stock returns, Event study

## Abstract

Despite blockchain’s potential to transform corporations by providing new ways of organizing business processes and handling information, extant research pays inadequate attention to how and under what conditions blockchain technology provides additional financial value for shareholders. Drawing on the efficient market hypothesis and signaling theory, we examined the relationship between firms’ blockchain use, development announcements, and stock market reactions. We used the event study methodology to analyze a sample of blockchain projects initiated by US firms between 2016 and 2019. The sample contains 114 firm-event observations. The findings show that the average abnormal return over a 2 days event period (including the day of the announcement and the day after the announcement) was positive. This positive stock market reaction is even more substantial when firms announce blockchain projects that focus on saving cost or time. Our findings also indicate that blockchain announcements tend to elicit more positive market reactions from smaller firms. We analyzed 249 firm-event observations containing firms from around the world and conclude that blockchain technology has a non-significant long-term impact on operating performance. The contingency approach adopted in our research provides advice for selecting the right mix of blockchain investment initiatives that is most suitable for a given organizational context.

## Introduction

Although the craze for Bitcoin has subsided, interest in blockchain (hereafter BC) technology has emerged. Given the potential of BC technology to transform traditional business operations and practices (Babich and Hilary 2020), top management teams have been pressed to decide whether to embrace BC. In 2020, Deloitte, an accounting firm, conducted a survey of 1488 senior executives from US and non-US companies. The survey results revealed that 39% of respondents say that their companies have already begun some BC deployment, and more than 36% say that their companies plan to spend at least US$5 million on BC over the next year. According to Grand View Research Inc., the global market for BC is expected to reach US$1431.5 billion by 2030, growing at a cumulative annual growth rate of approximately 86% from 2022 to 2030 (PRNewswire 2022).

Anecdotal evidence suggests that firms are generally motivated to invest in BC systems based on their expectations of improved operational and financial performance and increased business value (Carson et al. 2018; Flinders 2021; Warren and Deshmukh 2019). Nonetheless, practitioner reports and surveys show that regulatory issues and “uncertain return on investment” or “lack of clear return on investment” are among the top barriers to BC adoption and greater investment in BC (Bear et al. 2016; Carson et al. 2018; Matthew Budman 2019). Thus, approaching the value created by BC technology has become a primary demanding and pressing imperative (Xu et al. 2019). Accordingly, the primary goal of this study is to quantify the economic value associated with firm announcements regarding the implementation, development, or investment in BC systems.

Researchers commonly adopt the event study method as a first attempt to quantify the business value of novel information technology (IT) systems (Dehning et al. 2003a). An event study approach can measure the value that investors place on newly announced IT initiatives, based on expected future cash flows. Several previous studies have estimated the value of investing in IT systems by examining abnormal changes in stock prices and other performance metrics (Dos Santos et al. 1993; Hendricks et al. 2007). Specifically, this study employs short- and long-run event study methodologies to estimate the abnormal changes in stock returns and operating performance metrics following BC announcements.

Recent event studies examined the relationship between firms’ BC announcements and abnormal stock returns. For example, Cheng et al. (2019) investigated the stock market reaction to 82 US firms’ 8-K disclosures that mention BC-related activities. They found that speculative disclosures elicit a more favorable market reaction than non-speculative disclosures (firms’ strong commitment to BC). Cahill et al. (2020) report that the abnormal return on the announcement day is statistically significant at 5.0% for a global sample of 713 firm announcements. Their study also confirmed the overreaction to speculative announcements. Autore et al. (2021) provided evidence of significant shareholder value creation associated with the corporate adoption of BC. Klöckner et al. (2022) conducted an international event study to estimate the impact of BC initiatives on a firm’s market value. Their findings show that BC announcements have a significantly positive stock market effect. Using stock market data from Chinese A-shares, Chen et al. (2022) and Liu et al. (2022) investigated the impact of BC announcements on firm market value. They demonstrated that BC announcements result in positive market reactions. By contrast, Li et al. (2022) found that Chinese firms’ stock returns, return on assets (ROA), and volatilities decrease after being involved in the BC business.

Our study differs from previous studies in several ways. First, by conducting an in-depth content analysis of each announcement, we identify the distinct factors that influence stock market reactions to BC announcements. In particular, we determine the purpose of each BC project from the announcement and examine how it influences the market reaction, because the clear purpose of the BC project is thought to be especially important in determining its potential success (Felin and Lakhani 2018). Some recent studies have shown that abnormal stock returns induced by BC announcements are linked to various factors, such as the commitment level of a firm’s investment in BC (Cahill et al. 2020; Cheng et al. 2019), signaling credibility of BC announcements (Chen et al. 2022), ownership concentration (Li et al. 2022), industry’s research and development intensity, the country’s data restriction level (Klöckner et al. 2022), and the performance of Bitcoin (Cahill et al. 2020). To facilitate researchers’ understanding of the mechanisms through which BC influences firm value, we adopted a new perspective, in contrast to earlier work that mainly focused on relatively easily quantifiable factors. We extract cost/time-saving purposes from the text of BC announcements, which could be seen as a stronger positive signal. Second, the present study is unique in that it not only assesses the stock market reaction for firms announcing substantiated BC projects, but investigates the long-run operating performance effects of BC announcements. Changes in sales and operating income-based metrics for the sample firms are compared with a sample of control firms of a similar size and industry, which has not previously been examined in the BC literature. Third, we ensure the robustness of our empirical findings by complementing the main analyses based on a sample of US firms with a global sample. We use five different models to estimate abnormal stock returns.

A summary of the important findings and specific contributions to the literature are as follows: First, we integrate the efficient market hypothesis with signaling theory to provide a more comprehensive and complementary explanation of the impact mechanism of BC announcements on the stock returns of the announcing firm. Our theoretical perspective can serve as a useful foundation for future research on BC.

Second, we find a positive market reaction to BC announcements, consistent with previous research. Specifically, we find that our sample firms demonstrate significantly positive abnormal stock returns ranging from 7.26 to 7.43%, depending on the estimation model used. Our study adds to the discussion of how BC investments can create value for firms (Angelis and Da Silva 2019; Babich and Hilary 2020; Hastig and Sodhi 2020).

Third, previous research indicates that IT business value is sensitive to different internal and external contingencies; hence, we investigate when BC projects generate the most value. We show that BC announcements aimed at saving time or money can generate a 7.70% higher abnormal return rate than announcements without this goal. We also find that smaller firms’ BC announcements are associated with more positive market reactions.

Finally, this study builds on previous research on the use of accounting data to connect IT initiatives with firm performance (Dehning et al. 2007; Devaraj and Kohli 2003; Hasan et al. 2020; Hendricks et al. 2007; Johnson et al. 2007; McAfee 2002). Using operating performance metrics, we conducted a long-term event study. Our analysis of the long-run operating performance effects of BC announcements is important because investors and managers are likely to believe in estimates of economic impacts based on long horizons, as it provides them with a more complete picture of the economic implications of BC technology. However, the long-run event study results provide statistically weak evidence of improved operating performance for the sample firms after BC announcements.

Our findings have significant implications for both research and practice. In this study, we respond to previous research’s calls to more rigorously examine the efficacy of BC technology in business (Angelis and Da Silva 2019; Babich and Hilary 2019, 2020; Xu et al. 2019; Zhao et al. 2016). This study advances our understanding of how BC announcements affect corporate market values. Our research provides a necessary foundation for future theoretical studies (Dalenogare et al. 2018; Li et al. 2020). Furthermore, our research adds to the existing event study research that seeks to analyze stock market reactions to other new disruptive technologies (Goldberg et al. 2021; Lam et al. 2019). Providing empirical evidence of the positive effect of BC announcements on a firm’s market value highlights the general potential of BC technology for companies and contributes to a more nuanced understanding of BC’s current market acceptance. Our findings indicate that the market reaction to BC technology is context-dependent, which aids managers’ understanding of the conditions under which benefits can be maximized. For example, we recommend that practitioners begin BC implementation with applications that target inefficient and expensive business processes.

The remainder of this study is organized as follows. “Literature review, theoretical background, and hypotheses development” Section discusses the relevant literature, theoretical background, and hypothesis development. "Methodology" Section describes the methodology used in this study. "Results" and "Discussion" Sections present and discuss our results. Finally, “Conclusion” Section presents the study’s implications, limitations, and future research directions.

## Literature review, theoretical background, and hypotheses development

This section is divided into two sub-sections. The first subsection presents relevant literature and theories that discuss the link between BC announcements and firm market value. In the second section, we develop hypotheses on how the BC project’s purpose and firm size moderate the relationship between BC announcements and firm market value.

### Market reaction to BC announcements

Using BC, transactions between two parties can be recorded in a verifiable and permanent manner in an open, distributed ledger. (Iansiti and Lakhani 2017). The concept of BC was first introduced in 2008 by someone using the pseudonym Satoshi Nakamoto, who defined how cryptology and an open distributed ledger can be combined into a digital currency application (Nakamoto 2008; Xu et al. 2019). For a comprehensive survey of BC, especially in the subject areas of business and economics, see Xu et al. (2019).

The BC technology described above offers several benefits. Companies use BC technology to streamline business processes to save time and money. For example, Walmart’s BC experiment demonstrated that the time taken by the company to trace sliced mangoes from store to farm was reduced from 7 days to just 2.2 s. Companies can simplify their administration and document processing using BC. For example, the legacy camera brand Kodak introduced a BC-enabled document management system for businesses and governments to manage documents. According to Kodak, the BC system will result in cost savings of 20% to 40% owing to automated workflows and reduced human management of content, information, and documents (Kuhn 2019). The BC technology also enables financial and nonfinancial businesses to reduce the time between error detection and correction. For example, Italian banks tested a BC-enabled back office platform. The platform detects mismatched transactions automatically and eliminates the pain of reconciling mismatches, which take days or weeks to resolve, with banks emailing spreadsheets and calling each other. Mismatches on the BC platform can be immediately spotted and reconciled.

Unlike other technologies, BC systems enable business partners to transact directly, eliminating the need to rely on third parties. Thus, transactions are carried out without relying on explicit trust (of an intermediary), but on distributed trust, or so-called “meta-trust,” based on network consensus (Babich and Hilary 2020). Now that BC does not rely on a central unifying authority, it is a new revolution in every industry, from finance to retail to manufacturing firms, and practically any domain where mutual mistrust (between individuals, organizations, and organizations) is involved (Werbach 2018). The transaction information recorded on a BC is transparent and visible to users, as opposed to centralized systems, where users typically lack transparency or trust in the provider (Beck 2018).

The discussion above shows that BC technology offers a wide range of benefits to firms. Thus, from a signaling theory perspective, the announcement of a BC project helps a firm signal a commitment toward superior financial performance and operational efficiency (Chod et al. 2018). This reasoning is in concert with the efficient market hypothesis (Fama 1970) that new information about increased future cash flows resulting from the BC project should be immediately reflected in stock prices. In other words, investors should reward firms with increased market value as news about the BC project becomes available. In other words, investors interpret the benefits associated with BC positively, which, in turn, results in positive abnormal stock returns around the announcement date. The above discussion forms the basis of the following hypothesis:

#### Hypothesis 1 (H1)

BC announcements result in positive stock market reaction.

### Factors influencing the market reaction

Signaling theory also suggests that signal receivers, such as shareholders or investors, reward or penalize new information based on certain cues in the signal as well as the underlying qualities of the signaler (Connelly et al. 2011). Consequently, we posit that investors would consider a firm’s intention (toward BC) and characteristics when evaluating BC announcements. Accordingly, we propose two moderating factors that could augment or undermine stock market reactions to BC announcements.

#### The purpose of the BC project

Past research suggests that unplanned investment in technology may not be associated with improved firm performance (Das and Narasimhan 2001). Therefore, a clear purpose of the BC project is to generate significant value (Felin and Lakhani 2018). We examined how the intended BC project purpose is understood as a clear reason for developing, implementing, or investing in the BC, or more specifically, a specific business benefit that is supposed to affect the stock market reaction. Academic and practitioner research has identified several potential benefits of the BC technology (Carson et al. 2018; Islam et al. 2020; Wamba and Queiroz 2020). Papathanasiou et al. (2020) categorized BC-related business benefits into three broad categories: cost and time savings, improved monitoring and control, and the creation of a standardized platform. However, multiple BC project purposes or benefits are subject to various uncertainties and are not fully understood. We contend that the risks and uncertainties associated with the intended purpose or its characteristics (e.g., the quality of being measurable and the time frame to realize) can influence how a BC announcement is valued in the stock market. We investigated the effects of two types of BC project goals: *cost/time saving* and *others*, such as tracing physical objects or sharing data.

We propose that the *cost/time saving* purpose mentioned in the BC announcement signals a higher capability of being quantifiable and a higher probability of being materialized in the near future, potentially considered by investors (Carson et al. 2018; Zavolokina et al. 2020). Thus, by announcing a BC project whose intended purpose is *cost/time saving*, a firm will focus on short-term results to boost its stock price and benefit shareholders. Furthermore, we anticipate that investors will respond positively to another benefit associated with short-term oriented results; that is, they accelerate learning about the IT team’s previously unknown ability, allowing the announcing firm to condition its subsequent BC projects on this learning (Kazmi et al. 2021; Milbradt and Oehmke 2015; Thakor 2016).

In contrast, *other* BC project goals, such as making business processes more transparent, tracing physical objects, or sharing sensitive data among transacting parties, are less quantifiable and expected to be achieved over the long term (Papathanasiou et al. 2020; Zavolokina et al. 2020), and likely to attract limited investor attention. However, one could argue that investors may positively value the potential cost savings and revenue gains indirectly associated with *other* purposes. However, evidence suggests that, to realize most of the *other* purposes, the announcing firm must deal with particular challenges (Babich and Hilary 2020; Klöckner et al. 2022). For example, some suppliers may lack the necessary infrastructure and resources to participate in BC-based physical object tracing (Klöckner et al. 2022). Considering the realization of data storage and sharing purposes, the vulnerability of BC to external threats may create the risk of sensitive data loss or exposure (Wylde et al. 2022). Even though some of the *other* purposes of the BC project can create efficiency gains, we assume that investors are aware of the ongoing concerns and risks associated with *other* purposes and will therefore perceive the potential business value to be lower than for the *cost/time saving* purpose.

The above discussion leads to our second hypothesis:

##### Hypothesis 2 (H2)

Firms announcing BC projects intended to save costs and time produce higher positive stock returns than those announcing BC projects to achieve other purposes.

#### Firm size

The scale and scope of operations are referred to as the firm size. Prior research has yielded conflicting results. One viewpoint emphasizes that larger firms are more likely to benefit from new technological innovations in terms of market value because they have adequate resources and skilled IT teams. Simultaneously, another stresses that technological innovations should have a more substantial impact on the firm value of smaller firms because they can better adopt and effect change as they grow over time (Damanpour 1992).

It has been suggested that large firms dominate and control centralized sharing economies (Dedeoglu et al. 2020). For centralized sharing markets, small firms face numerous entry barriers (e.g., obtaining a license or regulatory clearance prior to operation, special tax benefits to existing firms, and strong brand identity). The BC-based platforms eliminate these barriers and open new business opportunities for small firms (Dedeoglu et al. 2020). For example, Net Element Inc., a small US-based financial technology company, established a BC-focused business unit in 2017 to invest in and disrupt the payment-processing industry by directly connecting merchants and consumers via its decentralized BC-based payment ecosystem. The current economic system is designed primarily for large firms, making it difficult for small businesses to obtain financing, scale operations, and recruit other ancillary services (Meijer 2019). We propose that BC enables small businesses to operate in previously unthinkable ways. For example, payments for goods or services from distant buyers and payroll to overseas employees have become simpler and can be completed at a fraction of the current cost thanks to BC. Consequently, it can assist small businesses in bringing products and transactional services to the market in a timely and cost-effective manner. Taking these advantages into account, we anticipate a stronger market reaction for smaller firms due to the higher salience of BC events in smaller firms and a weaker stock market reaction for larger firms due to a much smaller “surprise” element.

Accordingly, our third hypothesis is as follows:

##### Hypothesis 3 (H3)

The market reaction to BC announcements is significantly more positive for small firms than for large firms.

## Methodology

### Sample

To collect BC-related announcements, we searched the Business Wire (BW), PR Newswire (PRN), Reuters, and Wall Street Journal (WSJ) within the Factiva database covering 6 years, from January 1, 2014, to December 31, 2019. We performed a full-text search using a single keyword, BC, and searched for 22,200 articles. The first two authors then read all announcements independently to eliminate the announcement types listed in Step 2 of Table 12. To make the reading process more efficient, they first checked whether the firms mentioned in each announcement were publicly traded. For announcements involving publicly traded firms, they read the entire content of the announcement to decide whether to eliminate it based on the criteria listed in Step 2 of Table 12. After the above procedures, the first two authors had a discussion to check whether their samples contained the same announcements, and any inconsistencies were resolved. After reading and discussing, we eliminated 21,954 announcements, such as BC market analyses and BC consortium announcements.

A total of 246 announcements were retained after the aforementioned procedures (see Table 12 for details). We removed 40 announcements that contained confounding events. We also removed eight announcements because of insufficient firm data from the Center for Research in Security Prices (CRSP) or COMPUSTAT databases. Finally, we removed 94 announcements involving only non-US firms and used the remaining 104 announcements involving firms that publicly traded on US markets in our US sample analysis (see Step 3 in Tables 12 and 13). We have 114 firm-event observations in our short-term analysis because multiple firms could be involved in one announcement (see Table 13). The 114 firm-event observations involve 76 distinct US publicly traded firms because some firms conduct multiple BC projects. We use the daily stock returns of firms and the daily market returns of the Value-Weighted Index during the study period from the CRSP. Financial firm-level factors are obtained from COMPUSTAT.

### Event study method

The first step in executing an event study is to identify the exact event day accurately. All announcements in our sample first appear in BW, PRN, Reuters, or WSJ and include the time (EST: Eastern Standard time) and date of public release. We used the timestamp information on the announcements to determine the exact announcement date, as described by (Brandon-Jones et al. 2017; Hendricks et al. 2014). If the BC initiative announcement is made on a trading day during working hours (for US stock exchanges: 9:30 a.m.–4:00 p.m. EST), the calendar day is designated as the announcement date. If the announcement is made during stock market close (for US stock exchanges: after 4:00 p.m. EST) on a trading day or at any time on a non-trading day, we move the announcement date to the following trading day.

According to the efficient market hypothesis, stock prices quickly reflect new information about firms’ events (Fama and MacBeth 1973), and in line with previous research (Ba et al. 2013; Mishra et al. 2013), we concentrated our analysis on short-term abnormal returns (ARs) on the event date (day 0). However, to ensure that the market fully reacts to the event during the short horizon and no mispricing is reflected before or after the event, we also evaluate the event’s ARs in other periods, which include single- and multi-day event windows (i.e., trading day before the announcement date, trading day following the announcement date, trading day before the announcement date, announcement day, announcement day, trading day following the announcement date, and trading day before the announcement date to the trading day following the announcement date) as a robustness check.

The database data contain calendar days, but we are more concerned with their dates in relation to the event. We must translate calendar days into event days and redefine the subscript t of the return rate $R_{it}$*.* Consistent with the approach used in prior event studies by Brown and Warner (1985), $R_{i0}$ is defined as the return rate of stock i on the hypothetical event date, while t = 1 is the trading day following the announcement date, and t = − 1 is the trading day before the announcement date.

The next step is to calculate the ARs. The ARs is the gap between the actual and expected returns without an event. $AR_{it} = R_{it } - E(R_{it} |Y_{i} )$, where $AR_{it}$ is the ARs of stock *i* at time *t*, $Y_{i}$ denotes the information on day 0 except for the announcement, and $E\left( {R_{it} |Y_{i} } \right)$ is the normal or expected return if an event does not occur. We used several methods to estimate the normal return: $E(R_{it} |{\text{Y}}_{{\text{i}}} )$, including the market, mean-adjusted, market-adjusted, size-adjusted, and industry-adjusted models. Appendix B provides a brief introduction to the market model, the most widely adopted event study method (Ding et al. 2018; Sorescu et al. 2017), and other alternative models.

We proposed three hypotheses: For H1, we applied five different hypothesis tests to check whether the ARs on day *t* are significantly different from zero. We used two parametric test statistics, that is, the ordinary cross-sectional t-test and Patell’s test, whose null hypothesis states that the mean of the ARs within the event period is zero. The cross-sectional t-test is conducted by dividing the average event period ARs by its contemporaneous cross-sectional standard error. The cross-sectional t-test requires ARs to be uncorrelated across firms, which allows for event-induced volatility and serial correlation.

Patell’s test adjusts the weights of each abnormal stock return estimate by considering the estimate of variance of the residual stock returns. Unlike the cross-sectional t-test, Patell’s test requires the insignificance of event-induced variance.

We considered three nonparametric test statistics: the Corrado rank test, Wilcoxon signed-rank test, and generalized sign test. As noted by Jones et al. (2017), the Corrado rank test is a variation of the Wilcoxon signed-rank test. While the Wilcoxon signed-rank test relies on an overall cross-sectional ranking, the Corrado rank test is based on a firm-specific ranking of estimation and event-period ARs. Therefore, it is able to deal with the right-skewed nature of the ARs in a better manner.

These tests are often conducted in conjunction with a sign test to verify that a few firms do not drive the results (Boehmer et al. 1991). The generalized sign test is a variation of the binomial sign test. While the binomial sign test judges the proportion of positive and negative ARs against an assumed 50% split, the generalized sign test is adjusted to determine whether the percentage of positive ARs during the event period is significantly higher than that during the estimation period. The generalized sign test is able to deal with a non-symmetric distribution of ARs better. (Jones et al. 2017).

To test hypotheses H2 and H3, we regressed the log of ARs on day 0 ($$AR_{i0} + 1)$$ on the explanatory and control variables introduced below.

### Definition of variables

Regarding (H2), we have the explanatory variable cost/time Saving = 1 if the firm’s BC project aims to save cost/time and 0 otherwise. See Appendix D for the details of the coding process.

Regarding (H3), we have the explanatory variable firm size, which is measured as the natural logarithm of the firm’s total assets (in millions US$) in the fiscal year ending before the announcement date*.* In our US samples, the mean of total assets is US$$$1.77 \times 10^{5}$$ million, and the median is US$$$1.31 \times 10^{4}$$ million. Firms with total assets at or below the median value are small and those with total assets above the median value are large.

#### Control variables

Announcement-attribute controls: The first type of control variable is related to announcement attributes. We consider whether the announcement is the First Announcement (first announcement) concerning individual companies, because there may be several announcements concerning the sample company. For each company’s BC product maturity stage, one dummy variable (planning stage) is added: 1 if it is in the planning stage and 0 if it is in the implementation or completed stage. We also included a variable (User) to indicate that the company uses BC technology rather than developing it. Appendices C and D detail the coding procedures for the control variables.

*Firm-level controls*. The second type of control variable is firm characteristic. We used two variables: ROA, which is calculated as the ratio of operating income before depreciation to the book value of assets, and the debt-to-equity ratio (D/E), which is calculated as the firm’s book value of debt divided by shareholder equity. Firm-level control variables are based on information reported in the most recent fiscal year, ending prior to the announcement date.

Time-fixed effect: The third is the year fixed effect because our samples span 4 years, from 2016 to 2019. We used 2016 as the basis and create three dummy variables.

Industry fixed effects: The fourth type is at the industry level. We divided companies into eight groups using 2-digit Standard Industrial Classification codes (Table 13 in Appendix A), which include manufacturing, logistics and supply, wholesaling and retailing, depository institutions, non-depository credit institutions, insurance and real estate, services, and public administration. We begin with the service industry and create dummy variables for the other seven groups.

#### Fixed effects regression model

We estimate the following regression model:

1 $$Log(AR_{{i0}} + 1) = ~\beta _{0} + \beta _{1} Time/{\text{Cost}}~\,saving_{i} + ~\beta _{2} Firm\,~size_{i} + \overset{\lower0.5em\hbox{$\smash{\scriptscriptstyle\rightharpoonup}$}} {\beta } _{3}^{\prime } X_{i} + Year\,~dummies + Industry\,~dummies + \in _{i}$$

where $\mathop{X}\limits^{\rightharpoonup}$ includes the announcement attribute variables and firm-level variables of firm *i*. We use $AR_{i0} + 1$ to ensure that the value is positive, and take the logarithm of the dependent variables to reduce the effect of outliers. Here, we run a cross-sectional ordinary least squares (OLS) regression and use dummy variables to fix the time and industry effects.

## Results

This section presents the findings of this study. First, we offer the event study results, that is, the ARs associated with BC announcements (Table 3). Second, we show the ARs for different subgroups of events (Table 4). Third, we discuss our fixed effects regression model results to further investigate the relationship between the stock market reaction to BC initiatives and various moderators (Table 5).

### Descriptive statistics

Table 1 presents descriptive statistics of the explanatory and control variables. There are 56.14% BC announcements, whose purpose is to save time and cost. For the first time, 65.79% of BC announcements were made by firms. A few announcements (9.65%) were made in 2016.

**Table 1:** Descriptive statistics (*n* = 114)

*Explanatory variables*		*Industry sectors*	
Time/cost saving purpose	56.14%	Services	42.97%
Firm size^a^	8.80 (3.32)	Manufacturing	16.67%
*Control variables*		Logistics and supply	3.51%
First Announcement	65.79%	Wholesaling and retailing	6.14%
Planning stage	42.11%	Depository institutions	14.04%
BC user	42.98%	Non-depository institutions	10.53%
D/E	2.72 (5.45)	Insurance and real estate	4.39%
ROA	− 0.08 (0.42)	Public administration	1.75%
*Year variables*			
2016	9.65%		
2017	25.44%		
2018	33.33%		
2019	31.58%		

^a^Firm Size is the logarithm of total assets (in millions of US $). Numbers with percentage signs represent frequencies, numbers in brackets are standard deviations, and the remaining numbers are showing mean values. Mean and standard deviations are added only for non-dummy variables

Table 2 presents Spearman’s correlation coefficients. Spearman correlation can be used in many situations (e.g., linearity, heteroscedasticity, and normality) where other types of correlation do not hold true. Table 2 shows that the majority of the correlation coefficients are near zero, with the highest reported correlation coefficients being less than 0.5, indicating that no strong monotonic relationships exist between the variables.

**Table 2:** Correlation analysis (n = 114)

	1	2	3	4	5	6	7	8	9	10	11	12	13	14	15	16	17	18
1	1.00																	
2	0.18	1.00																
3	− 0.04	− 0.07	1.00															
4	− 0.16	− 0.25	0.04	1.00														
5	− 0.03	0.13	0.28	0.04	1.00													
6	− 0.04	0.34	− 0.16	− 0.04	0.01	1.00												
7	0.24	0.49	0.02	− 0.16	0.11	0.09	1.00											
8	− 0.01	0.20	0.11	0.09	0.08	0.08	0.09	1.00										
9	− 0.13	0.13	0.00	− 0.2	0.03	0.11	0.01	0.13	1.00									
10	− 0.05	− 0.35	0.08	0.05	− 0.04	− 0.15	− 0.23	− 0.35	− 0.41	1.00								
11	0.18	0.11	− 0.15	0.08	− 0.04	0.00	0.16	0.11	− 0.40	− 0.48	1.00							
12	− 0.32	− 0.29	0.02	0.01	− 0.05	0.00	− 0.26	− 0.29	− 0.05	0.23	− 0.15	1.00						
13	− 0.12	0.00	0.14	− 0.06	0.03	0.07	− 0.23	0.00	0.11	− 0.13	0.08	− 0.09	1.00					
14	− 0.29	− 0.06	0.03	0.23	0.08	− 0.07	− 0.08	− 0.06	− 0.15	0.05	− 0.02	− 0.11	− 0.05	1.00				
15	0.15	0.40	0.03	− 0.03	0.17	0.11	0.13	0.40	− 0.06	− 0.18	0.16	− 0.18	− 0.08	− 0.10	1.00			
16	0.02	0.02	− 0.05	− 0.06	0.17	− 0.01	0.03	0.02	0.39	− 0.06	− 0.23	− 0.15	− 0.07	− 0.09	− 0.14	1.00		
17	− 0.02	0.16	0.14	0.03	0.22	0.10	0.05	0.16	0.00	− 0.13	0.08	− 0.09	− 0.04	− 0.05	− 0.08	− 0.07	1.00	
18	− 0.02	0.14	0.10	− 0.11	0.02	− 0.02	0.04	0.14	− 0.08	0.19	− 0.09	− 0.06	− 0.03	− 0.03	− 0.05	− 0.05	− 0.03	1.00

1: Time/Cost saving, 2: Firm size, 3: First announcement, 4: Planning stage, 5: User, 6: Debt/Equity, 7: ROA, 8: 2016, 9: 2017, 10: 2018, 11: 2019, 12: Manufacturing, 13: Logistics and supply, 14: Wholesaling and retailing, 15: Depository institutions, 16: Non-depository financial institutions, 17: Insurance and real estate, 18: Public administration

### Event study results

Table 3 shows ARs from the market model (Panel A), the market-adjusted model (Panel B), the mean-adjusted model (Panel C), the size-adjusted model (Panel D), and the industry-adjusted model (Panel E). The results show that day 0 ARs are positive, with mean (median) ARs of 7.43% (0.00%) for the market model, 7.42% (0.21%) for market-adjusted model, 7.37% (0.21%) for mean-adjusted model, 7.26% (0.21%) for size-adjusted model, and 7.43% (0.21%) for industry-adjusted model. The median ARs are smaller in magnitude than mean, suggesting that stock returns are somewhat skewed to the right. The cross-sectional t test, the Patell’s test, and the Corrado rank test are significant at the conventional level for all models using day 0 as the event window, indicating that the mean is significantly different from zero. Meanwhile, the Wilcoxon signed rank test is significant at the 5% level for most of the models, except the size-adjusted model. In an event window [days 0 and 1], the median ARs are positive and the generalized sign test is significant at conventional levels for all models, indicating that the percent of firm-event observations having positive ARs are statistically significant. One day before (day − 1) the BC announcement, the abnormal stock returns are negative and significant on all reported measures for the market model and market-adjusted model, but some tests are insignificant in the other three models. As shown in Table 3, none of the five methods produces systematic evidence of significant abnormal stock returns on day 1, [days − 1 and 0], or [days − 1 to 1]. However, across five estimation methods, the cumulative abnormal returns (CARs) for the 2-days event period [days 0 and 1] are all positive and statistically significant at conventional levels, indicating that the stock market reaction is captured in the 2 days event period [days 0 and 1] as predicted by the market efficiency.

**Table 3:** Event study results

	Day − 1	Day 0	Day 1	[Days − 1 and 0]	[Days 0 and 1]	[Days − 1 to 1]
*Panel A: Market model (n* = *114)*						
Mean abnormal return (%)	− 0.95	7.43	− 0.51	6.46	6.91	5.95
Median abnormal return (%)	− 0.26	0.00	0.11	− 0.10	0.28	0.13
% Positive abnormal return	36.84	53.51	52.63	45.61	57.02	54.38
Cross-sectional t test	− 2.77**	2.41*	− 0.91	2.07*	2.33*	1.99*
Patell’s test	− 1.97*	9.92***	0.77	5.62***	7.56***	5.03***
Corrado rank test	− 2.39*	2.52*	0.70	0.09	2.28*	0.48
Wilcoxon signed rank test	− 2.64**	2.03*	− 0.22	0.43	2.26*	1.37
Generalized sign test	− 2.41*	1.13	0.95	− 0.54	1.88^a^	1.32
*Panel B: Market-adjusted model (n* = *114)*						
Mean abnormal return (%)	− 0.92	7.42	− 0.45	6.50	6.97	6.05
Median abnormal return (%)	− 0.31	0.21	0.06	− 0.04	0.37	0.13
% Positive abnormal return	38.60	55.26	52.63	48.25	62.28	50.88
Cross-sectional t test	− 2.59*	2.42*	− 0.77	2.12*	2.38*	2.08*
Patell’s test	− 1.99*	10.24**	0.82	5.83***	7.83***	5.24***
Corrado rank test	− 2.51*	2.39*	0.58	− 0.09	2.10*	0.26
Wilcoxon signed rank test	− 2.58**	2.40*	− 0.10	0.59	2.69**	1.51
Generalized sign test	− 1.90^a^	1.46	1.27	0.14	2.58**	0.89
*Panel C: Mean-adjusted model (n* = *114)*						
Mean abnormal return (%)	− 0.91	7.37	− 0.34	6.46	7.03	6.12
Median abnormal return (%)	− 0.41	0.21	0.10	− 0.22	0.42	0.42
% Positive abnormal return	35.96	56.14	56.14	45.61	62.28	56.14
Cross-sectional t test	− 2.47*	2.38*	− 0.58	2.08*	2.37*	2.07*
Patell’s test	− 1.41	6.30 ***	1.57	4.93***	8.13***	6.87***
Corrado rank test	− 2.41*	2.41*	1.04	0.00	2.44*	0.60
Wilcoxon signed rank test	− 2.74**	2.34*	0.69	0.71	3.31***	1.67^a^
Generalized sign test	− 2.70**	1.58	1.58	− 0.66	2.89**	1.58
*Panel D: Size-adj model (n* = *104)*						
Mean abnormal return (%)	− 0.91	7.26	− 0.17	6.34	7.08	6.17
Median abnormal return (%)	− 0.40	0.21	0.11	− 0.25	0.42	0.42
% Positive abnormal return	36.54	54.81	55.77	45.19	62.50	55.77
Cross-sectional t test	− 2.38*	2.17*	− 0.28	1.89^a^	2.20*	1.93^a^
Patell’s test	− 0.74	8.39***	1.37	5.41***	6.90***	5.21***
Corrado rank test	− 2.09*	2.07*	1.69^a^	− 0.01	2.66**	0.96
Wilcoxon signed rank test	− 2.45*	1.95^a^	0.83	0.16	3.21**	1.44
Generalized sign test	− 2.73**	0.99	1.19	− 0.97	2.56*	1.19
*Panel E: Industry-adj model (n* = *114)*						
Mean abnormal return (%)	− 0.84	7.43	− 0.27	6.59	7.16	6.32
Median abnormal return (%)	− 0.39	0.21	0.09	− 0.11	0.48	0.45
% Positive abnormal return	36.84	56.14	54.39	48.25	62.28	57.02
Cross-sectional t test	− 2.34*	2.40*	− 0.47	2.13*	2.42*	2.15*
Patell’s test	− 0.84	9.02***	1.38	5.79***	7.352***	5.52***
Corrado rank test	− 1.97*	2.55*	1.76^a^	0.42	3.05**	1.36
Wilcoxon signed rank test	− 2.50*	2.44*	0.67	0.84	3.50***	2.04*
Generalized sign test	− 2.69**	1.43	1.05	− 0.26	2.74**	1.61

The sample size in Panel D is 104 because we could not get the decile rankings information for 10 firms from Wharton Research Data Service

*p < 0.05, **p < 0.01, ***p < 0.001

^a^*p* < 0.10

In summary, the evidence from the 2 days [days 0 and 1] window indicates that BC announcements indeed generate positive abnormal stock returns for firms’ shareholders, supporting Hypothesis 1.

### Subgroup analysis

Table 4 shows the different subgroups’ average and median ARs on Day 0. We divided the US samples into five subgroups: timing effect (year), maturity stage, industry type, firm size, and purpose.

**Table 4:** Subgroup analysis results

All (n = 114)	Day 0			
	Mean	t-test	Median	Wilcoxon test
	*p*-value		*p*-value	
*ARs (%) by year*				
2016 (n = 11)	− 0.15	0.70	− 0.25	0.42
2017 (n = 29)	15.43	0.14	0.33^a^	0.06
2018 (n = 38)	5.60*	0.05	0.36*	0.02
2019 (n = 36)	5.36	0.26	− 0.24	0.99
*ARs (%) by maturity stage*				
Under planning (n = 48)	13.00^a^	0.06	0.26^a^	0.05
Implementation (n = 66)	3.45	0.10	− 0.04	0.20
*ARs (%) by industry type*				
Manufacturing (n = 19)	9.6^a^	0.09	0.39	0.31
Logistics and supply (n = 4)	29.51	0.40	− 0.78	0.72
Wholesaling and retailing (n = 7)	6.38	0.20	0.11	0.40
Depository institutions (n = 16)	10.29	0.34	0.29	0.57
Non-depository financial	0.64	0.45	− 0.11	0.94
institutions (n = 12)				
Insurance and real estate (n = 5)	− 0.25	0.83	− 1.17	0.50
Public administration (n = 2)	− 0.31	0.71	− 0.31	0.66
Services (n = 49)	6.85	0.22	0.26**	0.00
*ARs (%) by firm size*				
Small (n = 57)	15.06*	0.02	1.13***	0.00
Large (n = 57)	− 0.12	0.34	− 0.25^a^	0.06
*ARs (%) by purpose*				
Time/cost saving (n = 64)	8.54^a^	0.09	0.00	0.37
Others (n = 50)	6.10*	0.04	0.36*	0.03

*p < 0.05, **p < 0.01, ***p < 0.001

^a^*p* < 0.10

The industry codes are presented in Appendix A and Table 14. The t-test results may be misleading and unreliable because some subgroups are small, with fewer than 20 companies. Table 4 provides general information for different categories. As shown in Table 4, the ARs in 2017 was the highest for the 4 years. We compared ARs between BC projects with different maturity stages. The BC projects in the planning stage have higher ARs than those in the implementation stage. Comparing ARs between the different industry groups, we find that logistics and supply companies have the highest ARs. Furthermore, the results show that small firms have significantly higher ARs than large firms do. Finally, we considered the purposes of BC projects. The classification details of the types of purposes can be found in Appendix D. Firms that intended to achieve efficiency-oriented outcomes (e.g., time/cost saving) from BC technology have 2.44% higher ARs than those with other goals.

### Cross-sectional analysis results

We conducted regressions to test our hypotheses on the purpose of the BC project mentioned in the announcement and firm size. The dependent variable is the logarithm of the day 0 ARs for firm *i,* calculated using the market model described in Appendix B. Table 5 reports the results for the two models. In Model 1, we regressed the dependent variable on the control variables, and in Model 2, we regressed the dependent variable on the control variables and the hypothesized independent variables.

**Table 5:** Results of fixed-effects regression models

	Model 1	Model 2
	(SD)	(SD)
*Const*	− 0.05	0.07^a^
*Explanatory variables*	(0.03)	(0.04)
Time/cost saving		0.07*** (0.01)
Firm size		− 0.02** (0.00)
*Control variables*		
First announcement	0.05 (0.03)	0.04 (0.03)
Planning stage	0.05 (0.03)	0.04 (0.03)
User	0.03 (0.03)	0.03 (0.03)
D/E Ratio	− 0.01^a^ (0.00)	− 0.00 (0.00)
ROA	− 0.16 (0.12)	− 0.11 (0.11)
*Year fixed effects (2016)*		
2017	0.12* (0.04)	0.12** (0.04)
2018	0.01 (0.03)	− 0.02 (0.03)
2019	0.04* (0.01)	0.01 (0.02)
*Industry fixed effects (Service)*		
Manufacturing	− 0.02 (0.04)	0.00 (0.03)
Logistics	0.02 (0.09)	0.07 (0.08)
Wholesaling	− 0.03 (0.03)	0.02 (0.03)
Depository	0.02 (0.02)	0.08** (0.02)
Non-depository fin	− 0.10* (0.04)	− 0.09* (0.04)
Insurance	− 0.08* (0.02)	− 0.02 (0.03)
Public	− 0.02 (0.04)	0.07^a^ (0.03)
N	114	114
R^2^	0.27	0.33
Adjusted R^2^	0.16	0.21

*p < 0.05, **p < 0.01, ***p < 0.001

^a^*p* < 0.10

The time/cost saving purpose coefficient is positive and significant at the 0.01% level, indicating that stockholders value BC initiatives more for this purpose. This result supports hypothesis H2. The BC announcements with the goal of saving time and money can generate a 7.70%^1^ higher ARs rate than announcements without this goal. The firm size coefficient is negative and significant at the 0.62% level, indicating that small-company stocks react more positively to BC projects than large-company stocks, which supports H3. This coefficient indicates that a firm with one-third of the total assets of another firm would have 1.99%^2^ higher ARs if it announced the same type of BC project under similar conditions.

In conclusion, the regression results support H2 and H3: We also ran regressions with the dependent variable being a 2 days event window [days 0 and 1] CARs (see Table 16 in Appendix E). The regression results are consistent with the main results in Table 5.

### Robustness tests

Some additional analyses are performed in this section to support our primary results.

#### Event study results using regressions with event dummies

We ran an OLS regression with event dummy variables to support our results for Hypothesis 1. The analysis is based on the market model (see Appendix B), and we assumed the following linear relationship including entity effects:

2 $$R_{it} = \alpha_{i} + \beta { }R_{mt} + { }\gamma { }E_{it} + \epsilon_{it} ,$$

where $E_{it}$ is a dummy variable denoting the event period. For example, we analyzed the return rates at the event date (day 0), and $E_{it}$ is 1 if t is the event date for stock i. The covariance of the errors is clustered by entities (stocks). Table 6 presents the results of the event study based on regressions using event dummies. The event dummy coefficient one day before (day − 1) the BC announcement is significantly negative at the 5% level, the one on day 0 is significantly positive at the 5% level, and the coefficients in [days − 1 and 0], [days 0 and 1], and [days − 1 to 1] are significantly positive at the 5% level, which supports our main results for hypothesis 1.

**Table 6:** Results of regression models with event dummies

	Day − 1	Day 0	Day 1	[Days − 1 and 0]	[Days 0 and 1]	[Days − 1 to 1]
Market returns	1.01*** (0.03)	1.01*** (0.03)	1.01*** (0.03)	1.01*** (0.03)	1.01*** (0.03)	1.01*** (0.03)
Event dummy	− 0.01* (0.01)	0.08* (0.03)	− 0.01 (0.01)	0.03* (0.02)	0.04* (0.01)	0.02* (0.01)
N	136,976	136,976	136,976	136,976	136,976	136,976
Entities	67	67	67	67	67	67
R^2^	0.04	0.04	0.04	0.04	0.04	0.04
${\text{R}}^{2}$ Between	0.25	0.25	0.25	0.25	0.25	0.26
${\text{R}}^{2}$ Within	0.04	0.04	0.04	0.04	0.04	0.04

*p < 0.05, **p < 0.01, ***p < 0.001

#### Event study results using winsorized sample

We use winsorization to test our results for Hypothesis 1. Table 7 presents the event study results for ARs after 1% winsorization. The ARs in a 2 days event window [days 0 and 1] were still positive across the five models. The statistical tests are significant at the 5% level, except for the generalized sign test of the market model, which is significant at the 10% level in the 2 days event window [Days 0 and 1]. These findings indicate that Hypothesis 1 is supported after 1% winsorization.

**Table 7:** Event study results (Trim 1%)

	Day − 1	Day 0	Day 1	[Days − 1 and 0]	[Days 0 and 1]	[Days − 1 to 1]
*Panel A: market model (n* = *112)*						
Mean abnormal return (%)	− 1.05	3.69	− 0.31	2.64	3.38	2.33
Median abnormal return (%)	− 0.24	0.09	0.12	− 0.19	0.25	0.09
% Positive abnormal return	36.61	52.68	53.57	44.64	56.25	53.57
Cross-sectional t test	− 2.79**	2.52*	− 0.54	1.84 ^a^	2.38*	1.74 ^a^
Patell’s test	− 2.01*	4.32***	1.27	1.63	3.95***	2.07*
Corrado rank test	− 2.39*	2.70**	0.72	0.22	2.42*	0.59
Wilcoxon signed rank test	− 2.87**	2.01*	0.01	− 0.13	2.01*	0.68
Generalized sign test	− 2.36*	1.05	1.24	− 0.66	1.80 ^a^	1.24
*Panel B: market-adjusted model (n* = *112)*						
Mean abnormal return (%)	− 0.94	3.70	− 0.21	2.77	3.50	2.56
Median abnormal return (%)	− 0.29	0.17	0.11	− 0.07	0.40	0.07
% Positive abnormal return	38.40	54.46	53.57	47.32	61.61	50.70
Cross-sectional t test	− 2.59*	2.55*	− 0.38	1.94 ^a^	2.48*	1.93 ^a^
Patell’s test	− 1.81 ^a^	4.55***	1.45	1.94 ^a^	4.25***	2.42*
Corrado rank test	− 2.51*	2.56*	0.59	0.04	2.22*	0.37
Wilcoxon signed rank test	− 2.63**	2.15*	0.24	0.24	2.51*	1.23
Generalized sign test	− 2.27*	1.13	0.94	− 0.38	2.21*	0.19
*Panel C: mean-adjusted model (n* = *112)*						
Mean abnormal return (%)	− 0.92	3.57	− 0.10	2.65	3.47	2.55
Median abnormal return (%)	− 0.41	0.19	0.11	− 0.27	0.40	0.39
% Positive abnormal return	35.71	55.36	57.14	44.64	61.61	55.36
Cross-sectional t test	− 2.48*	2.45*	− 0.18	1.84 ^a^	2.44*	1.88 ^a^
Patell’s test	− 1.41	4.06***	1.99*	1.87 ^a^	4.28***	2.68**
Corrado rank test	− 2.41*	2.41*	1.05	0.00	2.45*	0.61
Wilcoxon signed rank test	− 2.64**	1.94 ^a^	0.87	0.24	2.94**	1.24
Generalized sign test	− 2.69**	1.47	1.84 ^a^	− 0.80	2.79**	1.47
*Panel D: size-adj model (n* = *102)*^b^						
Mean abnormal return (%)	− 0.93	3.09	0.10	2.16	3.19	2.26
Median abnormal return (%)	− 0.40	0.18	0.12	− 0.32	0.42	0.40
%Positive	36.27	53.92	56.86	44.12	61.76	54.90
Cross-sectional t test	− 2.37*	2.09*	0.17	1.49	2.15	1.62
Patell test	− 0.72	3.17**	1.84 ^a^	1.74 ^a^	3.54***	2.48*
Corrado rank test	− 2.09*	2.07*	1.86	− 0.01	2.78**	1.06
Wilcoxon signed rank test	− 2.47*	1.66 ^a^	1.19	− 0.18	2.96**	1.14
Generalized sign test	− 2.78**	0.78	1.38	− 1.20	2.36*	0.98
*Panel E: industry-adj model (n* = *112)*						
Mean abnormal return (%)	− 0.85	3.64	− 0.03	2.79	3.61	2.76
Median abnormal return (%)	− 0.39	0.21	0.11	− 0.18	0.46	0.43
%Positive	36.61	55.36	55.36	47.32	61.61	56.25
Cross-sectional t test	− 2.33*	2.51*	− 0.05	1.95^a^	2.56*	2.07*
Patell test	− 0.82	4.03***	1.83^a^	2.27*	4.14***	2.91**
Corrado rank test	− 1.96*	2.55*	1.92^a^	0.41	3.16**	1.45
Wilcoxon signed rank test	− 2.52*	2.18*	1.01	0.54	3.27**	1.76^a^
Generalized sign test	− 2.74**	1.23	1.23	− 0.47	2.55*	1.42

*p < 0.05, **p < 0.01, ***p < 0.001

^a^p < 0.10

^b^We could not get the decile rankings information for 10 firms from Wharton research data service

#### Event study results using global samples

As a robustness check, we also use global samples to test our hypotheses on the BC project purpose and firm size. There are 249 firm-event observations and 184 distinct firms in the global sample (see the description in "Sample" section, and Tables 12 and 13 in the Appendix for details on sample development), because some firms are involved in multiple events. In the global sample, we use the country of origin to distinguish firms. Among the 184 distinct firms, 64 firms are headquartered (“located”) in US; 18, Chinese firms; 12, Japanese firms; 11, Canadian firms; 8, Indian firms; 8, UK firms; 8, French firms; and the remaining firms are from other countries.

Table 8 shows the outcomes of the two models. The dependent variable in these models is the abnormal log return from the market model. As there are 184 distinct firms in the global sample, industry categories must be adjusted by adding two more types: agriculture and public administration. Table 8 shows that we obtained consistent results using the global sample compared to the US sample. The coefficient of time/cost saving is still significantly positive and that of firm size is negative at the 1% level. The results based on the global sample support H2 and H3:

**Table 8:** Regression model for global sample

	Model 1	Model 2
*Const*	− 0.01 (0.02)	0.06 (0.03)
Time/Cost Saving		0.03* (0.01)
Firm size		− 0.01** (0.00)
First Announcement	0.02 (0.01)	0.02 (0.01)
Planning Stage	0.03^a^ (0.02)	0.02 (0.02)
User	− 0.01 (0.02)	− 0.01 (0.01)
D/E Ratio	− 0.00 (0.00)	− 0.00 (0.00)
ROA	− 0.00 (0.00)	0.00 (0.00)
*Year fixed effects* (2016)		
2017	0.05* (0.02)	0.05^a^ (0.03)
2018	0.01 (0.02)	− 0.00 0.02
2019	0.02^a^ (0.01)	0.02 (0.01)
*Industry fixed effects* (Service)		
Agriculture	− 0.05*** (0.01)	− 0.05*** (0.01)
Manufacturing	0.01^a^ (0.00)	0.02* (0.01)
Logistics	0.01* (0.01)	0.03** (0.01)
Wholesaling	0.014 (0.01)	0.04* (0.02)
Depository	0.04^a^ (0.02)	0.06* (0.02)
Non-depository fin	− 0.01 (0.01)	0.00 (0.01)
Insurance	0.00 (0.02)	0.02 (0.02)
Public	− 0.00 (0.01)	0.04^a^ (0.02)
N	249	249
Adjusted	0.08	0.14

*p < 0.05, **p < 0.01, ***p < 0.001

^a^p < 0.10

### Long-term event study results for a global sample using operating performance measures

This section reports the results of using the one-to-portfolio matching method. Appendix F describes the process and criteria used to select the control firms. For each performance measure, we report two sets of results in Tables 9 and 11. Each Table shows the mean and median changes in one of the three operating performance metrics (two profitability-based and one sale-based). The first set of results (Panel A in each Table) depicts the year-to-year performance change in the sample firms or BC-adopting firms in comparison to the control firms over the 4-years period starting from year − 1 to year + 2. The second set of results (Panel B in each Table) compares the performance of sample and control firms over several longer time intervals to reflect different patterns of performance change across firms.

**Table 9:** Control adjusted percentage changes in operating income

Event Year	Obs	Mean	Median	Standard Dev	t-test	Wilcoxon test
*Panel A: changes in performance on an annual basis*						
Year − 1	97	− 0.27	− 0.18	2.37	− 1.12	1057***
Year 0	98	− 0.30	− 0.06	3.58	− 0.84	1832*
Year + 1	89	8.86	0.00	90.40	0.93	1873
Year + 2	53	− 10.19	0.05	65.74	− 1.13	687
*Panel B: changes in performance over varying time period*						
[Years − 1 and 0]	97	− 0.59	− 0.15	4.32	− 1.35	1414***
[Years 0 and 1]	89	8.53	− 0.04	90.37	0.89	1697
[Years 1 and 2]	53	− 11.49	− 0.01	66.12	− 1.27	584
[Years 0–2]	53	− 11.50	− 0.04	66.47	− 1.26	485*

Wilcoxon test shows the statistics of the signed-rank sum

*p < 0.05, **p < 0.01, ***p < 0.001

Table 9 presents the percentage changes in operating income. Panel A of Table 9 implies that the median changes in the operating income of the sample firms are negative and statistically significant in years − 1 and 0. The other median and mean changes were not significant. Panel B of Table 9 shows that the median changes in operating income are negative and statistically significant in [years − 1 and 0] and [years 0–2]. The other median and mean changes were not significant. Overall, Table 9 shows that the operating income of the sample firms did not statistically significantly affect BC adoption, except in year 0, the median change of which is statistically negative.

Table 10 reports the percentage changes in sales over 1 year (Panel A) and over longer time periods (Panel B). Panel A shows that annually over 3 years, the mean (median) change in sales of the BC adopting firm is lower than that of control firms in Years − 1, 0, and + 1. However, for firms involved in BC projects, the annual mean change in sales is not significant in Year + 2, but the median change in sales is significantly lower. Except for a few exceptions, Panel B shows that the mean (median) changes in sales over longer time periods are all negative and highly significant. Interestingly, the magnitude of negative median changes has reduced from − 0.16 in [years − 1 and 0] to − 0.16 and − 0.09 in [years 0 and 1] and [years 1 and 2] respectively, indicating an improvement in the performance of the sample firms. A similar (but small in magnitude) downward-trend can also be observed in mean changes over the same years.

**Table 10:** Control adjusted percentage changes in Sales

Event Year	Obs	Mean	Median	Standard Dev	t-test	Wilcoxon test
*Panel A changes in performance on an annual basis*						
Year − 1	77	− 0.44	− 0.04	1.62	− 2.38*	861***
Year 0	79	− 0.43	− 0.10	1.66	− 2.29*	687***
Year + 1	71	− 0.47	− 0.03	2.32	− 1.73^a^	870*
Year + 2	41	− 0.39	− 0.04	2.09	− 1.19	268*
*Panel B changes in performance over varying time period*						
[Years − 1 and 0]	77	− 0.87	− 0.16	2.30	− 3.33***	605***
[Years 0 and 1]	71	− 0.89	− 0.16	2.83	− 2.64**	522***
[Years 1 and 2]	41	− 0.75	− 0.09	2.39	− 2.01^a^	200**
[Years 0–2]	41	− 1.03	− 0.22	3.81	− 1.72^a^	153***

Wilcoxon test shows the statistics of the signed-rank sum

*p < 0.05, **p < 0.01, ***p < 0.001

^a^*p* < 0.10

Table 11 summarizes the performance of the sample companies relative to that of the matched portfolios of the control firms. In Table 11, the sample firms’ adjusted performance is computed by subtracting the mean (median) performance of the matched portfolio of the control firms from the performance of our sample firms. A positive portfolio-adjusted performance indicates that the sample companies outperformed the portfolios of control firms. According to Panel A of Table 11, in terms of mean ROA, the sample firms did not outperform their matched portfolios in any of the years from − 1 to + 2. However, in year + 2, the sample firms outperformed their matched portfolios in terms of the median ROA at the 5% level. Note that the negative mean (median) differences in year − 1 have changed to positive differences or are smaller in magnitude in the years following the announcement of the BC project, providing hope to BC technology optimists.

**Table 11:** Sample firm’s mean and median adjusted Return on Assets (ROA) based on portfolio of control firms

Event Year	Obs	Mean (%)	Median (%)	Standard Dev	t-test	Wilcoxon test
*Panel A changes in performance on an annual basis*						
Year − 1	97	− 1.04	− 0.26	6.62	− 1.55	1637**
Year 0	98	− 1.2	0.01	9.26	− 1.35	2468
Year + 1	89	− 0.49	− 0.07	18.50	− 0.25	1831
Year + 2	53	0.28	0.30	5.04	0.41	974*
*Panel B changes in performance over varying time period*						
[Years − 1 and 0]	97	− 2.34	− 0.28	15.10	− 1.52	1826
[Years 0 and 1]	89	− 1.88	0.09	0.17	− 1.05	1910
[Years 1 and 2]	53	0.06	0.11	5.71	0.07	957*
[Years 0–2]	53	− 0.37	0.27	6.34	− 0.43	873

Wilcoxon test shows the statistics of the signed-rank sum

*p < 0.05, **p < 0.01, ***p < 0.001

In contrast to the analysis summarized in Panel A of Table 11, which compares the performance of the sample companies to the matched portfolios of control firms at particular points in time, Panel B of Table 11 compares the improvement rates of the sample companies and their respective matched portfolios of control firms. Panel B summarizes the portfolio-adjusted rate of improvement across alternative time periods by calculating the differences in the mean (median) changes between a sample firm’s ROA and a control firm’s ROA over the same period. A positive portfolio-adjusted rate of improvement indicates that the sample firms’ rate of improvement is greater than that of their matched portfolios of control firms over the specified period. According to Panel B, the mean changes in ROA in all years are negative, but insignificant in three of the four intervals. However, we must note that the negative mean difference between [years 0 and 1] is smaller in magnitude than the difference between [years − 1 and 0], and is positive (though insignificant) between [years 1 and 2]. These observations provide weak evidence of the positive effects of BC technology on the long-term operating performance of the sample companies. Over a longer interval, we find that in Panel B the only significant and positive median abnormal ROA appears in [years 1 and 2].

## Discussion

This study empirically assesses the shareholder-value impact of BC use and its development announcements. We find that the stock market reacts positively to BC announcements, thus supporting H1. Depending on the model used to estimate ARs, the mean abnormal return on the event day ranged from 7.26 to 7.43%. The positive stock market reaction to BC technology announcements is consistent with recent studies investigating other disruptive technologies. For example, by evaluating announcements of 3D printing implementation, Lam et al. (2019) and Goldberg et al. (2021) documented positive stock returns. Another recent study by Teo et al. (2016) reported that business analytics announcements (as a tool for making smart business decisions) generate positive ARs. Son et al. (2014) found a positive stock market reaction to announcements of cloud-computing initiatives. Specifically, the positive stock market reaction to BC announcements reported in our study is consistent with that of Cahill et al. (2020) and Cheng et al. (2019), who explore BC and Bitcoin announcements. Our results are also consistent with those of Autore et al. (2021), Chen et al. (2022), Klöckner et al. (2022), Liu et al. (2022), who investigated the stock market reaction to substantiated BC announcements. However, the theoretical perspective of this study is different from that of Autore et al. (2021), Klöckner et al. (2022), Liu et al. (2022). Autore et al. (2021) study builds on the strategic disclosure literature. Specifically, they study how public firms respond to disruptive new innovations such as BC in terms of their disclosure behaviors, as well as how investors respond to such disclosures. Although Klöckner et al. (2022) and Liu et al. (2022) consider the value of substantiated BC initiatives from the IT business value and technology management perspectives, respectively, they do not discuss the effect of BC announcements and contextual factors on stock prices based on signaling theory. To some extent, the theoretical framework of Chen et al. (2022) is close to our perspective. However, they mainly focused on signaling the credibility of firms’ fundamentals rather than the attributes of the BC announcement text. Specifically, they found that high-tech firms with more technological reserves could be viewed as more credible and produce more positive stock returns than non-high-tech firms when releasing BC announcements. Prior IT studies have leveraged the signaling theory in a wide area of research. For example, Nishant et al. (2017) studied the signaling effects of green IT announcements, which result in positive abnormal stock returns and an increased share trading volume. Among the potential signaling mechanisms that have been studied within the context of BC, the majority involve BC-based fundraising (Chanson et al. 2020; Fisch 2019; Jiang et al. 2022) and supply chain transparency (Chod et al. 2018). However, they did not examine the signaling mechanisms in the context of capital markets. We differentiate between these studies by applying signaling theory in the context of signaling to equity investors.

Specifically, we examine the signaling value of the attributes of BC announcements (such as the purpose of the BC project) and firms. These findings indicate that the financial market recognizes the potential of BC as an emerging digital tool capable of increasing operational efficiency, which is directly and indirectly related to improved firm performance. We further investigated whether the positive impact of BC announcements on firm stock returns is affected by the purpose of the BC project and firm size and provide evidence to support H2 and H3.

Consistent with H2, BC announcements have a greater positive impact on a firm’s market value if the purpose of the BC project is *saving costs/time*, implying that shareholders should consider the characteristics of the BC project’s purpose when evaluating a firm’s investment in BC. This finding is consistent with previous event studies that emphasized the significance of the purpose or benefits associated with IT initiatives (Dehning et al. 2003b; Dos Santos et al. 1993). In particular, this result supports Son et al. (2014) finding that the announcement of a cloud computing initiative has a greater positive impact on the market value of a firm with informed operational benefits (e.g., cost savings) than that with informed strategic benefits. Our findings support those of Klöckner et al. (2022), who found that the positive stock market reaction to BC announcements is weaker when the BC is used for strategic purposes, such as tracing physical objects or sharing sensitive data. Managers frequently use cost and transaction-time efficiencies to justify the results of BC systems (Matthew Budman 2019). From the lens of signaling theory, one possible explanation for our finding is the perception that efficiency-oriented BC benefits are achievable in the near future, and that measuring these benefits is easier than measuring other benefits. This provides shareholders with a positive investment signal and makes the market reaction to an efficiency-oriented announcement more positive. It also indicates that the market is currently more interested in the operational potential (e.g., cost savings) of BC than in strategic or long-term benefits because the commercialization of BC applications has recently expanded. The market needs more time to understand and trust BC’s strategic value (Klöckner et al. 2022). Thus, our findings suggest that announcements of BC projects intended to achieve efficiency-oriented objectives (e.g., costs/time-saving) are strong signals.

Interestingly, our finding is different from that of Liu et al. (2022), who used 143 BC announcements from an emerging market. Liu et al. (2022) find that strategic-level BC announcements exhibit a greater stock price increase than operational-level announcements. The difference in the findings may be due to a significant proportion of Liu et al. (2022) sample of BC announcements (32%) belonging to the acute phase of the COVID-19 pandemic, which has accelerated the pace of digital transformation and changed how companies approach digital technology. After the outbreak of the pandemic, firms used digital technologies, including BC, to achieve transformational and sustainable goals (Aysan et al. 2021; Soto-Acosta 2020). Thus, in the post-pandemic era, a stronger positive stock market reaction to strategic-level BC announcements could be expected. Another reason might be the difference in a firm’s approach toward BC in emerging and developed countries. As noted by Liu et al. (2022), Deloitte’s 2019 Global Blockchain Survey shows that 73% of Chinese firms regard BC as their strategic focus. Our finding also contradicts Chen et al. (2022) results, which show that the textual sentiment in Chinese firms’ BC announcements requires a longer period to be captured by investors and that the stock markets have no significant response in the short term. This difference might be due to the possible differentiation in investors’ information perception caused by imperfect information channels in Chinese capital markets (Chen et al. 2022).

Consistent with H3, BC announcements demonstrate a more positive stock market reaction toward small firms, reflecting the important role that a signaler’s characteristics play in the effect of BC announcements on stock value. In contrast to Klöckner et al. (2022) and Liu et al. (2022), we find that small firms have higher positive abnormal stock returns after BC projects are announced. However, our finding regarding firm size is consistent with that of Cahill et al. (2020). Moreover, our findings are consistent with the emerging view that BC-enabled decentralized applications and platforms are good solutions for small businesses facing numerous challenges (Ko et al. 2018). For example, access to adequate financing is a major business problem in small businesses. Among all participants, BC-based decentralized credit platforms alleviate information asymmetry and credit rationing issues through a decentralized consensus and information distribution. Thus, our findings suggest that the announcement of a BC project by a small firm sends strong positive signals of their commitment and increases shareholders’ confidence in the successful implementation or development of a BC (Liu et al. 2022).

Finally, we discuss the long-term event study results, which were not supported by our research. Our results show that BC adoption and development announcements may not necessarily improve operating performance. This finding may be due to the short analysis period. The literature on performance measures suggests that accounting-based performance indicators are lagging measures that represent a firm’s performance over a specific period (e.g., a fiscal year). This finding suggests that it takes a relatively long period for the impact of a firm’s strategy to be reflected in accounting-based performance measures, especially when technology implementation is involved. For instance, Hendricks et al. (2007) explored the impact of the implementation of enterprise systems on operating performance measures over a 5-years period. Such an approach is not applicable to our research, as 68% of the BC projects were announced in 2016–2018, suggesting that our sample size will drop considerably if a 5-years investigation period is used.

## Conclusion

Since the inception of BC, attention to BC’s potential in transforming firms’ operations is relatively limited compared to the vast majority of studies on cryptocurrency (Fang et al. 2022; Sebastião and Godinho 2021). In this study, we use the event study method to examine the stock market reaction to 114 firm-event observations of US-listed firms from 2016 to 2019. The event study results show that BC announcements have a significantly positive stock market reaction. According to our cross-sectional regression analysis, stock returns are more pronounced for firms announcing BC projects aimed at achieving efficiency-oriented BC benefits and for small firms. These findings support our efficient market hypothesis and signaling theory. As discussed below, the empirical evidence documented and the theoretical perspective adopted in our study have important implications for research and practice.

### Implications

#### Implications for research

This study has several implications. First, previous event studies have frequently employed the resource-based theory to explain the relationship between IT investment announcements and firm performance. In this study, we show that by combining the efficient market hypothesis and signaling theory, these two theories complement each other in some cases and provide holistic explanations for the findings. To the best of our knowledge, this is the first study to examine the economic value of BC using the efficient market hypothesis and signaling perspectives. This theory allows us to connect firms’ BC activities with their stock return performance. We believe that our theoretical perspective provides a solid foundation for future BC research.

Second, the theoretical framework used in this study expands our understanding of the relationship between BC announcements and stock returns by considering not only the direct impact of BC on stock returns but the indirect moderating role of the BC project’s purpose and firm characteristics. Our findings suggest that investors favor BC announcements when the BC project and the announcing firm demonstrate certain characteristics. Future research could look into other BC projects and firm characteristics as signals (Chod et al. 2018). This could broaden BC research beyond proverbial questions such as “whether BC results in positive payoffs” by “digging deeper” into conditions where payoffs are likely to be boosted or reduced (Teo et al. 2016).

Finally, the widespread acceptance of signaling theory, as well as the importance of perception of returns from BC announcements, emphasizes the importance of examining the short-term market value impact from a psychological rather than an organizational theory perspective. Psychological theories, such as prospect theory and construal level theory (Xu and Yiu 2015) along with signaling theory, could provide a new understanding of the phenomenon.

#### Implications for practice

This study’s findings have several critical managerial implications. First, this study assures top executives that investment in BC could have a positive impact on market value.

Second, this study illustrates the importance of the BC project’s purpose. Our results suggest that firms seeking positive payoffs in terms of market value should implement or develop BC in areas that improve time and cost savings. For example, in the service sector, it is critical to automate clearing and settlement, trade finance, and cross-border payment transactions to reduce transaction processing time and costs. Other industries should use BC to streamline and automate their day-to-day economic exchanges and business operations, such as invoice generation and reconciliation, customs clearance, insurance claim processing, and property title transfer.

Third, our findings suggest that managers of small firms should recognize the positive market value effects of using and developing the BC technology. Several decentralized BC platforms, such as the We.Trade Blockchers project and the Alastria Blockchain Ecosystem, specifically designed for small firms, have been launched in recent years. We recommend that executives of small firms take a bold step toward exploring such innovations. By understanding these boundary conditions and firm-specific contexts, top executives can better plan BC announcements to have a greater impact on firm market value.

### Limitations

This study has several limitations. For starters, this study disregards long-run stock price reactions to BC announcements. According to existing literature, to obtain a more comprehensive estimate of an event’s economic impact, one must examine stock price performance in the post-announcement period (Hendricks and Singhal 2005). The long-run stock price effect of BC is significant because managers and investors are more likely to trust estimates of the economic impact based on long horizons because they provide a more complete picture of the economic implications of BC technology.

Second, although we controlled for variables, such as industry sector, BC project maturity stage, and firm’s role in the BC project, we did not consider other factors, such as the firm’s innovation capability, IT intensity, or competitive environment. Third, the timing of BC announcements may influence stock market effects. As the industry and shareholders become more aware of the benefits and drawbacks of a BC, their reactions to BC announcements may change. The findings of this study are based on a small sample of BC announcements from the US market (2016–2019); therefore, some of our findings may not be generalizable to future event studies. As previously discussed, firms’ strategic-level BC announcements may receive more positive stock market reactions than operational-level BC announcements in the post- pandemic era.

Finally, firms’ adoption of BC technologies should be a self-selection process that may raise endogeneity concerns. This study did not address potential endogeneity issues.

### Directions for future research

We discuss three promising directions for future research: First, we do not investigate the risk effects of BC announcements. Previous research indicates that implementing advanced IT systems can reduce risks. For example, Rubin and Rubin (2013) report a significant reduction in stock return volatility after intelligent business system deployment. Future research could examine which types of BC projects announced by companies result in high risk in terms of stock volatility. Future studies could integrate the risk perspective with signaling theory to understand whether announcements as signals reduce or enhance market risk.

Second, future research could include the firm’s innovation capability or IT intensity and the competitive environment as moderating factors of the stock market reaction to BC announcements, because these moderating variables have the potential to influence the strength of the signal.

Third, the moderating variables used in our analysis present the BC project and firm characteristics (e.g., the purpose of the BC project and firm size) at a specific time point based on BC announcements. Such cross-sectional analysis is suitable for understanding relationships rather than causality. Therefore, future research should employ cutting-edge estimation techniques with longitudinal datasets to test the causality between contextual factors and the market value of BC.

Finally, the sample size was small. However, this is consistent with previous studies. ﻿With the increasing adoption of BC by firms in the post-pandemic period, data availability should be less of a limitation.

## Appendices

### Appendix A: announcements filtering procedures and industry classification

Table 12 summarizes our announcements filtering procedures, and Table 13 shows the size of different samples used in our analysis. Table 14 shows our industry classification using SIC codes.

**Table 12:** Announcements selection procedures

Steps	Description	Number of announcements
Step 1	*Announcements that matched keyword “blockchain”*	22,200
Step 2	*Announcements eliminated:*	21,954
	Announcements that contain information about BC market analysis (size, growth, trends and forecast) Announcements that discuss regulation issues regarding BC adoption Announcements that contain firms’ announcements regarding awards and recognition for inventing BC-related IT solutions, appointing BC technology experts, and BC consortia Announcements that only involve private firms Announcement on cryptocurrency (e.g., Bitcoin) Other irrelevant announcements (firms’ clarification on their BC business, BC conferences and seminars, company notices on financial results, etc.) Announcements that were not the earliest if announcements having the same content were published by multiple publications	
	*Announcements retained*	246
Step 3	*Announcements eliminated*	142
	Announcements affected by confounding company news (e.g., change in CEO, increase/decrease in dividend, earnings surprises, etc.)	40
	Announcements by firms missing information on CRSP/COMPUSTAT	8
	Announcements involving only non-US firms	94
	Announcements retained	104

**Table 13:** Samples used in data analysis

Samples	Sample size
US sample of firm-event observations	114
Global sample of firm-event observations	249

**Table 14:** Industry classification

Industry level	SIC codes	US	Global
Agriculture and natural resources	0001–1999	0	10
Manufacturing	2000–3999	19	40
Logistics and supply	4000–4999	4	18
Wholesaling and retailing	5000–5999	7	11
Depository institutions	6000–6099	16	53
Non-depository institutions	6111–6163, 6211–6289, 6711–6799	12	32
Insurance and real estate	6311–6399, 6512–6553	5	11
Services	7000–8999	49	71
Public administration	9000–9999	2	3

### Appendix B: computation of abnormal returns using market model, mean-adjusted model, market-adjusted model, size-adjusted model, and industry-adjusted model

The market model assumes a linear relationship between stock and market returns:

3 $$R_{it} = \alpha_{i } + \beta_{i} R_{mt} + \epsilon_{it} ,$$

where $R_{mt}$ is the market return on day t, $\alpha_{i }$ is the intercept, is $\beta_{i}$ the slope of the relationship, and $\epsilon_{it}$ is the error term for stock *i* on day t. We estimate these two parameters using an OLS regression on a 200-days estimation window from day − 210 to day − 11. The window ends two weeks (10 trading days) prior to day 0 to avoid the announcement’s impact. Then the expected returns, abnormal returns and cumulative abnormal returns are:

4 $$E(R_{it} |X_{i } ) = \hat{\alpha }_{i} + \hat{\beta }_{i} R_{mt} ,$$

5 $$AR_{it} = R_{it} - E(R_{it} |X_{i } ),$$

6 $$CARs_{i} \left( {t_{1} ,t_{2} } \right) = \mathop \sum \limits_{{t = t_{1} }}^{{t_{2} }} AR_{it} ,$$

where $\hat{\alpha }_{i}$ and $\hat{\beta }_{i}$ are estimated parameters of OLS regression, and CARs stands for cumulative abnormal returns.

For robustness check, we can add the event dummy variables (${\text{E}}_{{{\text{it}}}} = 1$ if it’s a date for the BC announcement of this announcement, 0 otherwise) to Eq. (3) (see Eq. (2) in “Event study results using regressions with event dummies” Section), then run OLS regression on panel data, which contains observations on multiple stocks over multiple time periods. We can test our hypothesis 1 by checking the significance of the coefficients for event dummies (${\upgamma }$). However, this method assumes that the BC announcement has a similar impact on stock returns of different firms, i.e. ${\upgamma }$ is homogeneous across firms. This assumption is not true in the real world. Therefore, we only present results in “Event study results using regressions with event dummies” Section. The other approach is to run OLS regression on each stock separately and then test the coefficients of the event dummies. We adapt the abnormal returns in the primal analysis, since we want to study which firm-level factors contribute to better performance with BC, i.e. H2 and H3.

The mean-adjusted model calculates the average return over the estimation window as the expected return for specific security, which means

7 $$E\left( {Y_{i} } \right) = \frac{1}{200}\mathop \sum \limits_{j = - 210}^{ - 11} R_{ij} ,$$

Similarly, the market-adjusted model uses the returns on the market index over the event period as the estimated normal return, which is equivalent to

8 $$E\left( {Y_{i} } \right) = R_{mt} ,$$

where $R_{mt}$ is the market daily return on Day *t*.

Both the size-adjusted model and industry-adjusted model construct a firm i’s matching portfolio $M_{i }$ and use the daily returns of this portfolio over the event period as the estimated normal returns. The size-adjusted model uses the size deciles to find matched companies, while the industry-adjusted model uses the industry deciles. $M_{i }$ should not contain any firm which have announced BC initiatives. We download return data for size deciles and Fama–French 5-industry portfolios from Kenneth R. French data library and calculate the expected returns by:

9 $$E\left( {Y_{i} } \right) = \mathop \sum \limits_{{j \in M_{i} }}^{ } W_{ij} R_{jt} ,$$

where $W_{ij}$ is the weight of stock j in the portfolio $M_{i }$. We present the results of equally-weighted portfolios in Table 3.

### Appendix C: coding details for BC project maturity stage

We divide BC projects maturity stage into the planning stage and implementation stage. BC projects in the planning stage refer to plans for BC projects intended to start at some time point in the future. It could be establishing collaboration, a plan for developing BC solution, acquiring a company developing BC solution/platform, or establishing a BC-focused business unit or subsidiary. BC projects in the implementation stage refer to an already initiated BC project in the form of a BC solution/platform development such as in-progress prototypes, pilot projects, or testing. Alternatively, BC products have been successfully launched and are available to use or have been used. BC project types in different maturity stages and corresponding keywords appearing in the announcements are listed below.

- BC projects in the planning stage:
- Establish collaboration:

Join (join forces on/to; agree to develop jointly); Agreement (a new agreement; announce a strategic agreement; enter into/sign/conclude an agreement); Partner/team up (join partners; has teamed up to develop; a strategic partnership; (would) team up to explore/create; partnered/partnership to build); MoU (sign a MoU; enter into MoU) Alliance (the formation of a strategic alliance); Joint venture (enter into JV and service agreement; establishment of a joint venture collaboration (announce a new collaboration; are embarking on an industry-first collaboration; collaborate to launch; has collaborated to build).

- Plan:

Plan to use; will develop; will explore; intend to use; are/is launching a project; are/is launching/preparing a pilot program; will launch a trial; or aims to launch.

- Acquisitions/investment:

Acquisition/lab/unit/start-up (acquire 51% of a group; purchase of a BC company; the launch of/establish a BC-focused business unit; the launch of/open a BC lab; will establish BC lab; established a division; a new wholly-owned subsidiary; will establish a subsidiary; set up a BC unit; agrees to set up a new start-up).

- BC projects in implementation stage:

Prototype (have built/developed a prototype; is providing a prototype version; demonstrate a BC prototype). Pilot (a pilot launch; the launch of a BC pilot; completed a pilot; completed a pilot; collaborate on a pilot program).

Test (is testing; team up to test; has successfully tested; the platform was tested; successfully tested; started testing a BC-based platform; has tested and validated; passes the testing).

Working/launching (is working with; are launching the first BC application; is developing a program using its platform; are collaborating; are building; began a project; has partnered on a Proof of Concept).

Developed/Completed/Available (conducted/completed/developed/built/produced a BC practice/solution/network/platform;pioneered thefirst BC solutions complete development/final testing; availability of a BC solution; successful completion of a BC- tracked mobile advertising campaign; demonstrate the feasibility; the placement of; have started a blockchain trading platform; a new platform is released; is officially accessible; the BC solution can now use or is accessible; has delivered).

Launch (launch of BC service/platform/solution/system/network/product/application; the market launch; has launched; successful launch; was launched; unveiled a new digital payment platform).

Implementation (has processed/implemented/completed/replicated/introduced; start to run BC trades; is using; to commercialize; has become the first fledged trade; made the first deposit on the platform; perform the world's first trade finance).

### Appendix D: coding details for the purpose of BC project

We used Factiva data base to collect firms’ announcements related to BC technology. A final sample of 104 news articles was selected for analysis. We assumed that in each BC project announcement, the company (s) had mentioned their primary purpose: a clear reason for developing, implementing, or investing in BC; or, more specifically, certain business advantages that are supposed to achieve. Initially, 20% of the total 104 announcements were randomly selected and completely analyzed by all the authors. Disagreements were observed and discussed until consensus was reached (Yang et al. 2012). This process led to the development of a coding scheme. The details of the coding scheme are as follows:

First, each article covering the announcement was assigned a unique identification number.

Second, after reading each article's full text, one or more paragraphs containing potential purposes were identified.

Third, relevant sentences were identified after careful reading of each paragraph.

Fourth, each selected sentence was deeply analyzed to identify the exact phrase (s) that contain the purpose of the project.

Fifth, from each qualified phrase, we selected single word or compound words (e.g., transparency, cut cost) that represent the project’s primary purpose.

Sixth, we standardized most of the original keywords to avoid a broad set of keywords. For standardization, we tried to use symbolic terms (e.g., cost reduction) that frequently appeared in the sample announcements and the BC literature.

Finally, we placed standardized keywords into appropriate categories. We classified firms most frequently appeared purposes into two main categories.

The first category named as “saving cost/time” includes the following standardized keywords: automation, complexity reduction, cost reduction, digitalization, disintermediation, efficiency, optimization, productivity, simplification, and time saving. BC-enabled automation, digitalization, complexity reduction, disintermediation, optimization, and simplification are good reasons for saving cost and time.

In the second main category, “Others,” transparency, security, track-ability, and transformation are at the top, while there are also some less frequently occurred keywords in this group.

Out of 104, 4 announcements have not mentioned a clear purpose, and 77% of total announcements have mentioned 1 to 3 different purposes per announcement. Using the above coding scheme, two authors read all of the articles selected for the final sample and executed the coding job individually. Finally, to cross-validate the coding results, two more judges were brought in and disagreements were resolved on a majority rule basis.

Table 15 presents the subgroup analysis of day 0 abnormal returns for all six major purposes (i.e., time/cost Saving, Security, Transparency, Traceability, BC Infrastructure, and transformation).

**Table 15:** Subgroup analysis for all purposes

Purpose	Mean (%)	T-test	Medium (%)	Wilcoxon test
	*p* value		*p* value	
Time/cost saving (n = 64)	8.54^a^	0.10	0.00	0.37
Security (n = 25)	1.73	0.18	0.29*	0.02
Transparency (n = 23)	0.10	0.70	− 0.18	0.65
Traceability (n = 19)	0.18	0.61	− 0.09	0.94
BC infrastructure (n = 10)	8.35	0.25	1.56	0.24
Transformation (n = 7)	7.44	0.19	0.83 ^a^	0.09

One announcement may have more than one purposes

^a^*p* < 0.10

*p < 0.05

### Appendix E: cross-sectional analysis for cumulative abnormal returns (days 0 and 1)

This section presents the cross-sectional analysis results using a 2 days window [days 0 and 1] CARs as the dependent variable. The coefficient for time/cost saving purpose is positive and significant at the 0.01% level. The coefficient of firm size is negative and significant at the 0.90% level. The regression results for the CARs [days 0 and 1] also support both H2 and H3.

See Table 16.

**Table 16:** Results of fixed effects regression models

	Model 1 (SD)	Model 2 (SD)
*Const*	− 0.06 (0.03)	0.06 (0.04)
*Explanatory variables*		
Time/cost saving		0.07*** (0.00)
Firm size		− 0.02** (0.01)
*Control variables*		
First announcement	0.05 (0.03)	0.03 (0.03)
Planning STAGE	0.06 ^a^ (0.03)	0.05 ^a^ (0.03)
User	0.05* (0.02)	0.05 (0.02)
D/E ratio	− 0.01 (0.00)	− 0.00 (0.00)
ROA	− 0.13 (0.12)	− 0.08 (0.11)
*Year fixed effects (2016)*		
2017	0.14* (0.04)	0.15*** (0.03)
2018	0.01 (0.03)	− 0.03 (0.03)
2019	0.04^a^ (0.02)	0.01 (0.02)
*Industry fixed effects (Service)*		
Manufacturing	− 0.02 (0.04)	0.01 (0.03)
Logistics	0.01 (0.08)	0.07 (0.07)
Wholesaling	− 0.04 (0.03)	0.03 (0.03)
Depository	0.01 (0.01)	0.07** (0.01)
Non-depository fin	− 0.13** (0.02)	− 0.12* (0.02)
Insurance	− 0.09** (0.02)– 0.00 (0.03)	− 0.02 ^a^ (0.01)
Public		0.09 ** (0.03)
N	114	114
R^2^	0.26	0.32
Adjusted R^2^	0.14	0.20

*p < 0.05, **p < 0.01, ***p < 0.001

^a^*p* < 0.10

### Appendix F: long-term event study method

The multi-step process we used to establish our control firms is as suggested by Barber and Lyon (1996). We use specific standards to match the announcement company with other companies and compare their operating performance. In the matching procedure, we use the below six steps to find comparable companies:

- *Step 1* Companies should have headquarters in the same country.
- *Step 2* Not only should we compare their performance in the same year, but they should also have the same fiscal year end month.
- *Step 3* Companies should belong to the same industry type. We identify those firms with the same 2-digit SIC code as the company involved in BC announcement.
- *Step 4* Companies should have similar accounting performance in the previous year. We choose those whose ROA in year − 2 (the fiscal year before the study period) is within 0.70–1.30 times (or 70–130%) of the sample firm’s ROA.
- *Step 5* Companies should be of comparable size. Comparable companies’ total assets should be greater than one-third (33%) of the sample firms’ total assets but less than three times the sample firms’ total assets (or 300%). In other words, a sample firm’s comparison group consists of all firms with assets ranging from 33 to 300% of the sample firm’s total assets. In monetary terms, if a sample firm has total assets of US$100 million, a control firm with total assets of US$33 million to US$300 million should be included in the control group.
- *Step 6* Matched companies should not be involved in any BC announcement. After matching, 98 sample companies have at least one comparable company.

We calculate average performances in each matching group and compare them with the sample company’s performance. For operating income and net sales, we use percentage changes as performance estimation:

$$Percentage\,\, change_{t} = \frac{{I_{t} - I_{t - 1} }}{{\left| {I_{t - 1} } \right|}}$$

where $I_{t}$ represents current year’s operating income or net sales and $I_{t - 1}$ is the previous year’s operating income or net sales.

For ROA, we just use its actual changes.

Finally, we calculate the abnormal operating performance for a firm using:

$$Abnormal \,\,operating \,\,performance = OP - \widehat{OP}$$

where $OP$ is the actual operating performance of the company with BC initiatives and $\widehat{OP}$ is the expected operating performance if BC initiative had not occurred. We use the average performance of the matched group in event period to estimate the expected operating performance if BC initiative had not occurred. All companies don’t have any BC announcement in the event window.

## Data Availability

The datasets used and/or analyzed during the current study are available from the corresponding author upon reasonable request.

## Abbreviations

BC: Blockchain
IT: Information technology
H1: Hypothesis 1
H2: Hypothesis 2
H3: Hypothesis 3
BW: Business wire
PRN: PR newswire
WSJ: Wall Street Journal
ARs: Abnormal returns
CRSP: Center for Research in Security Prices
EST: Eastern Standard Time
ROA: Return on assets
D/E: Debt-to-equity
SIC: Standard Industrial Classification
CARs: Cumulative abnormal returns
OLS: Ordinary least square

## Footnotes

1 From Eq. (1), we have derived the formula $\widetilde{{{\text{AR}}}} - {\text{AR}} = \left( {{\text{e}}^{{\upbeta }} - 1} \right) \times \left( {{\text{AR}} + 1} \right)$, where $\widetilde{{{\text{AR}}}}$ is the abnormal return with Time/Cost Saving and AR is the one without this. From Table 4, we can estimate that the mean of AR (other purposes) is 6.10%. Thus, the difference between two abnormal returns is $\left( {\exp \left( {0.07} \right) - 1} \right) \times \left( {6.10\% + 1} \right) = 7.70\%$.

2 Similarly, from Eq. (1), we get the formula $AR_{1} - AR_{2} =$ $\left( {e^{\beta 1 \times \Delta } - 1} \right) \times (AR_{2} + 1)$, where ${\Delta }$ is the difference of log total asset. Then from Table 2, ${\text{AR}}_{2}$ is 7.43%. Thus, the difference is 1.99%.
